# First trimester fetal aortic Doppler for hemoglobinopathies

**DOI:** 10.4274/tjod.24381

**Published:** 2016-06-15

**Authors:** Cihan Çetin, Selim Büyükkurt, Ebru Dündar Yenilmez, Mehmet Özsürmeli, Mete Sucu, Cansun Demir

**Affiliations:** 1 Çukurova University Faculty of Medicine, Department of Obstetrics and Gynecology, Adana, Turkey; 2 Çukurova University Faculty of Medicine, Department of Medical Biochemistry, Adana, Turkey

**Keywords:** Hemoglobinopathies, Thalassemia, sickle cell anemia, Prenatal diagnosis, Doppler ultrasonography

## Abstract

**Objective:**

To evaluate fetal aortic Doppler for the prenatal diagnosis of hemoglobinopathies in the first trimester of pregnancy.

**Materials and Methods:**

Between January and November 2014, a total of 108 patients were enrolled in the study. The couples were carriers of either alpha/beta thalassemia, sickle cell disease or combined carriers of these and were admitted to Çukurova University Faculty of Medicine, Department of Obstetrics and Gynecology Prenatal Diagnosis Center. One hour before the chorionic villus sampling (CVS), patients were evaluated using fetal aortic Doppler. Pulsatility index, peak systolic velocity, and heart rate were noted.

**Results:**

There were no statistically significant differences in Doppler indices between different groups of CVS results when compared with the healthy controls.

**Conclusion:**

Fetal aortic Doppler investigation was found to be ineffective for the prenatal diagnosis of hemoglobinopathies.

## INTRODUCTION

A hemoglobinopathy is a genetic defect that results in abnormal structure of one of the globin chains of the hemoglobin molecule. There are various types of defects known today. They are single gene disorders and generally inherited in an autosomal recessive pattern. The most common are beta/alpha-thalassemia and sickle cell disease.

These diseases have different prevalences around the world. Beta-thalassemia is particularly prevalent among Mediterranean people, whereas alpha-thalassemia is common in sub-Saharan Africa, the Mediterranean Basin, the Middle East, South Asia, and Southeast Asia. Sickle cell disease is found most prevalently in sub-Saharan Africa, tribal regions of India, and the Middle-East^(1)^. Therefore, these hemoglobinopathies are important health problems for Turkey because of its geographic location.

The heterozygote carriers of these diseases show some degrees of anemia, whereas homozygote mutation carriers exhibit significant health problems. They require intermittent blood transfusions and have many complications such as increased incidence of infections, cholelithiasis (gallstones), cholecystitis, and growth problems during childhood. Carriers also face complications related to treatment such as excess iron load and related complications in thalassemias and other complications of frequent blood transfusions. Bone marrow transplantation is the only known cure for these diseases.

Prenatal diagnosis is very important for carrier couples. Early prenatal diagnosis may provide the chance of early termination for these couples during the first trimester. Current approaches for prenatal diagnosis include invasive [chorionic villus sampling (CVS), amniocentesis or cordocentesis] and non-invasive diagnosis with fetal DNA in maternal plasma^(2,3,4)^.

Alterations in fetal blood parameters in hemoglobinopathies may cause changes in fetal Doppler parameters. Therefore, prenatal diagnosis using Doppler ultrasound may be another noninvasive method for the carriers. In this study we aimed to evaluate the indices of fetal aortic Doppler for the non-invasive diagnosis of hemoglobinopathies.

## MATERIALS AND METHODS

One hundred eight patients were enrolled in this prospective study between January 2014 and November 2014. All patients and their husbands were carriers of either alpha/beta thalassemia, sickle cell disease or combined carriers of these. They were admitted to Çukurova University Faculty of Medicine, Department of Obstetrics and Gynecology Prenatal Diagnosis Center for the prenatal diagnosis of hemoglobinopathies. They all had CVS during 11 and 14 weeks of their pregnancy. Gestational age was based on crown-lump length (CRL). Ethical Committee Approval was obtained from Çukurova University Clinical Research Ethics Committee (Report no: 28/14) and all subjects gave informed consent to participate in the study.

**Figure 1:**
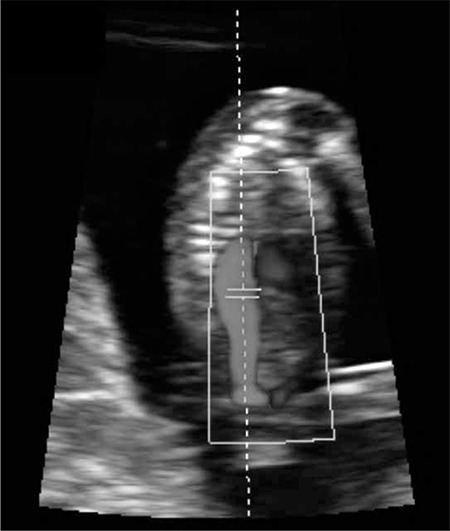
First trimester fetal aortic Doppler

One hour before the CVS, patients were examined using a GE Voluson 730 Pro-ultrasound machine with a convex volumetric transabdominal (RAB 4-8 MHz) probe by a single experienced operator. In order to achieve an insonation angle of <30 degrees, fetuses were examined in a position where the fetal aorta was perpendicular to the probe (Figure 1). The magnification of the image was such that the fetus occupied the whole screen. The range gate was set at 2 mm. Pulsatility index, peak systolic velocity (PSV), and heart rate (HR) were noted.

After the ultrasound examination, CVS was performed. Fetal karyotype was also determined for all fetuses. Patients were divided into groups by the results of the hemoglobinopathy status. Therefore, the operator was blinded to the patients’ groups.

Student’s t-test was used for normally distributed data, and the Mann-Whitney U test was used to compare non-normally distributed data. The p value of <0.05 was considered as statistically significant. Statistical analyses were performed using Statistical Package for the Social Sciences (SPSS) version 15 (SPSS, Inc., Chicago, IL).

## RESULTS

The mean age of the patients was 28.34±5.2 years (range, 17-40 years). The mean gestational age was 12±0.8 weeks (range, 11-14 weeks) and the mean CRL of the fetuses was 59.90±9.6 mm (range, 41-85 mm). There was no statistically significant differences between the groups regarding the mean age of the women and CRL of fetuses based on CVS results. Eight women were Rh negative but none were isoimmunized. Hemoglobinopathy status of the couples is given in Table 1. All CVS procedures were completed without complication. The karyotypes of all fetuses were normal. Hemoglobinopathy results and the Doppler indices of the fetuses are summarized in Table 2.

There were no statistically significant differences in Doppler indices between the different groups of CVS results compared with the healthy (Hemoglobin AA) controls. Comparisons were made in subcategories of gestational weeks (11, 12, 13, and 14 weeks), because Doppler indices normally differ with advancing gestational age. Furthermore, there was no statistically significant difference between subgroups as for fetal HR (mean fetal HR=165.01±3.1 beat/min).

All patients whose fetuses were diagnosed as having sickle cell disease and beta-thalassemia major decided to terminate pregnancy, all were terminated without complication using sublingual misoprostol.

## DISCUSSION

Fetal arterial and venous Doppler evaluations are used effectively in different trimesters. For instance, fetal ductus venosus Doppler is found effective in screening for aneuploidies during the first trimester, whereas it is used for fetal well-being from the mid-trimester. The other most commonly used Doppler evaluations include umbilical, uterine, and middle cerebral arteries; each has a different purpose such as fetal well-being, preeclampsia screening, and detection of fetal anemia. Of these, middle cerebral artery PVS is used for the detection of fetal anemia but it is technically used after the 18^th^ week of gestation^(5)^.

Fetal anemia can be expected in hemoglobinopathies, especially in homozygous alpha-thallassemia^(6)^. During the first trimester, fetal aortic Doppler is found to have significantly different indices in fetuses with homozygous alpha-thalassemia^(6)^. In a recent study by Karateke et al.^(7)^ ductus venosus Doppler was also found to have significantly different indices during the first trimester in fetuses with beta-thalassemia and sickle cell disease^(7)^.

To the best of our knowledge, this is the first study to investigate fetal aortic Doppler for the prenatal diagnosis of beta-thalassemia and sickle cell disease during the first trimester. We chose fetal aorta for screening because it can be easily visualized during this period. We were able to visualize the fetal aortic blood flow in all patients with transabdominal probe. No transvaginal evaluation was needed.

We found no significant difference in fetuses with beta-thalassemia and sickle cell disease in arterial Doppler parameters during the first trimester compared with the healthy fetuses. Therefore, current fetal aortic Doppler was not found effective for the prenatal diagnosis of these hemoglobinopathies.

Fetal hemoglobin is a tetramer composed of two copies of each of two different peptide chains. The type of chains determine the type of hemoglobin produced. Adult hemoglobin consists of two beta and two alpha chains; however, during embryonic and fetal stages there are various types of hemoglobins. The first fetal hemoglobins are produced in the yolk sac and are called hemoglobin (Hb) Gower 1 (two zeta chains and two epsilon chains), Hb Gower 2 (two alpha chains and two epsilon chains), and Hb Portland (two zeta chains and two gamma chains)^(8)^. Erythropoiesis then moves to the liver, where Hb F (two alpha and two gamma chains) is produced. Finally, at around 11 weeks, normal Hb A is produced by fetal bone marrow and progressively increases in fetal blood as the fetus matures^(9)^. The final adult version of the alpha chain is produced exclusively by 6 weeks and there are no functional alternative versions thereafter^(10)^. If an alpha gene mutation or deletion occurs, there is no alternate alpha type chain that can substitute to form functional hemoglobin. In contrast, at least two versions of the beta chain (gamma and delta) remain in production throughout fetal life and beyond. In the case of a beta gene mutation or deletion, these two other versions of the beta chain often continue to be produced, which results in Hb A2 or Hb F and substitute for the abnormal or missing hemoglobin^(10)^. The reason that we found no hemodynamic change in fetuses with hemoglobinopathy was probably because of these substitute hemoglobins. In fetuses with hemoglobinopathy, substitute hemoglobins (Hb A2 and Hb F) conceal the disease during fetal life, but the disease manifests after birth. However, fetuses with alpha gene defect may even manifest during fetal life if all three or four genes are affected, because of the lack of substitute chains^(11)^. We had no fetuses with alpha thalassemia major (Hb Barts or Hb H disease) in our study.

The main limitation of our study is the small number of patients in some subgroups of patients, such as alpha-thalassemia and combined heterozygotes of hemoglobinopathies.

## CONCLUSION

Currently, invasive prenatal diagnostic tests such as CVS, amniocentesis or cordocentesis are the most widely used invasive tests for hemoglobinopathies(12). Non-invasive diagnostic tests using fetal DNA in maternal plasma shows promising results; however, they are expensive and not yet widely available around the world. Therefore, non-invasive prenatal diagnostic tests like Doppler ultrasound investigations should still be continued.

## Figures and Tables

**Table 1:**
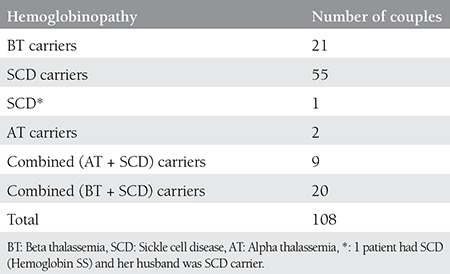
Hemoglobinopathy status of the couples

**Table 2:**
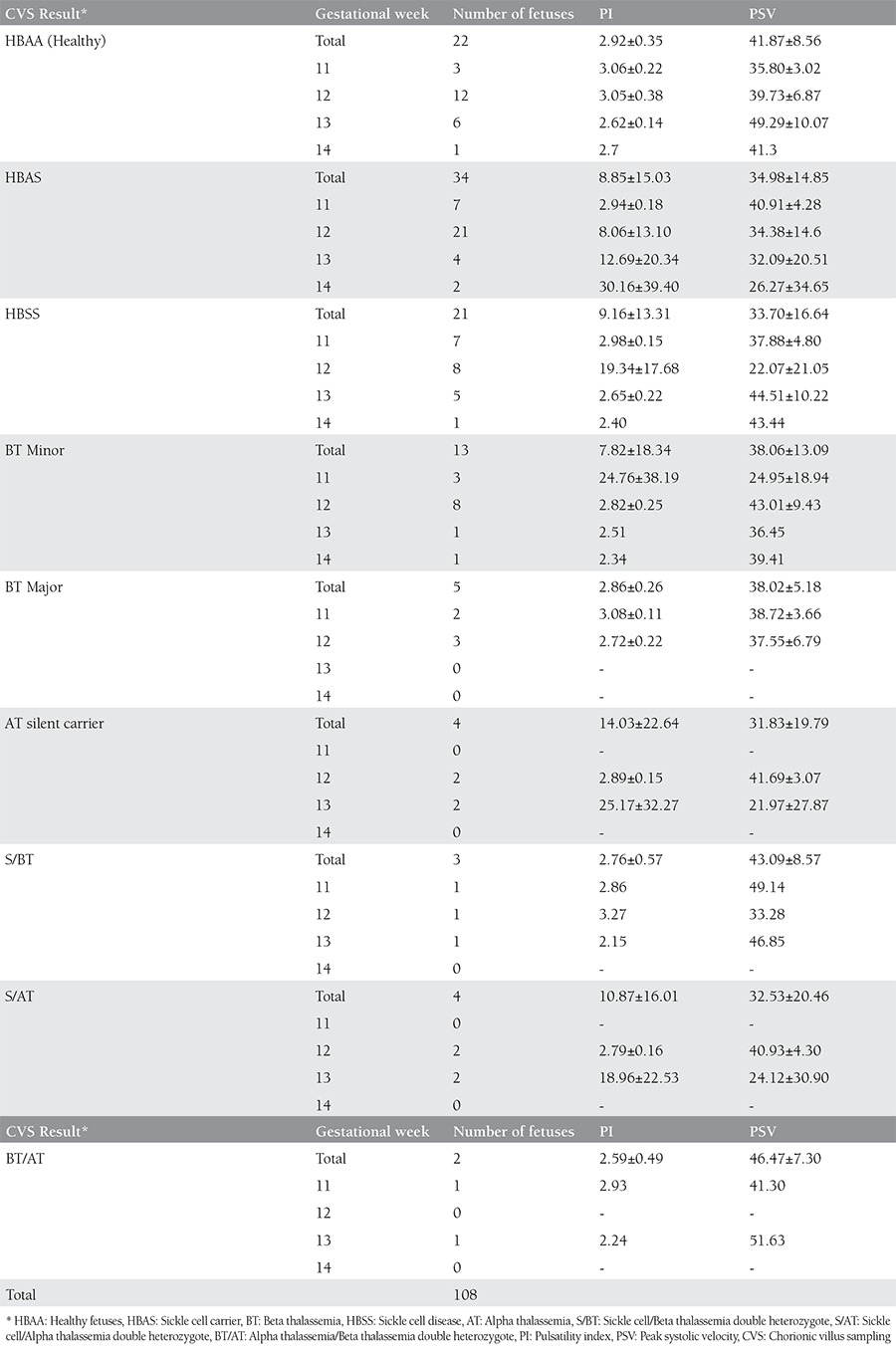
Chorionic villus sampling results and doppler indices of fetuses
